# A Survey on Design, Actuation, Modeling, and Control of Continuum Robot

**DOI:** 10.34133/2022/9754697

**Published:** 2022-07-25

**Authors:** Jingyu Zhang, Qin Fang, Pingyu Xiang, Danying Sun, Yanan Xue, Rui Jin, Ke Qiu, Rong Xiong, Yue Wang, Haojian Lu

**Affiliations:** ^1^ State Key Laboratory of Industrial Control and Technology, Zhejiang University, Hangzhou 310027, China; ^2^ Institute of Cyber-Systems and Control, The Department of Control Science and Engineering, Zhejiang University, Hangzhou 310027, China; ^3^ Department of Plastic Surgery, Sir Run Run Shaw Hospital, Zhejiang University of Medicine, Hangzhou 310016, China

## Abstract

In this paper, we describe the advances in the design, actuation, modeling, and control field of continuum robots. After decades of pioneering research, many innovative structural design and actuation methods have arisen. Untethered magnetic robots are a good example; its external actuation characteristic allows for miniaturization, and they have gotten a lot of interest from academics. Furthermore, continuum robots with proprioceptive abilities are also studied. In modeling, modeling approaches based on continuum mechanics and geometric shaping hypothesis have made significant progress after years of research. Geometric exact continuum mechanics yields apparent computing efficiency via discrete modeling when combined with numerical analytic methods such that many effective model-based control methods have been realized. In the control, closed-loop and hybrid control methods offer great accuracy and resilience of motion control when combined with sensor feedback information. On the other hand, the advancement of machine learning has made modeling and control of continuum robots easier. The data-driven modeling technique simplifies modeling and improves anti-interference and generalization abilities. This paper discusses the current development and challenges of continuum robots in the above fields and provides prospects for the future.

## 1. Introduction

Inspired by the tentacles of elephant trunks, snakes, and octopuses, continuum robots use a series of continuous arcs structurally rather than skeletal structures to generate bending motion [1], with flexibility, lightweight, inherent safety, scalability, and potential for low-cost parts. This design approach was initially applied primarily in industrial scenarios such as large-scale grasping, movement, and positioning [2] and even urban search and rescue operations in confined environments [3]. With the development of some advanced materials, the scale of the continuum robot is getting smaller and smaller [4]. With its soft characteristics, it has been widely used in the field of medical surgery.

In addition to the robots composed entirely of continuum medium, super-redundant robots with many discrete links are also regarded as continuum robots. The earliest work can be located in 1967, when Anderson [5] studied the first tensor arm composed of stacked plates that can generate motion by stretching tendons. After that, there is a lot of research on super-redundant continuum robots [6–8]. A typical example is the super-redundant robot developed by CardioARM [9], which is a highly redundant serpentine arm for cardiac ablation. In recent years, parallel designs of continuum robots have also been developed. This kind of robot uses multiple elastic rods connected in parallel arrangement and has higher accuracy and stiffness than the serially connected continuum robot [10–12]. Some typical examples of parallel continuum robots include the multispine snake robot proposed by Ding et al. [13], Festo bionic tripod manipulator [14], and Stewart-Gough continuum design [15].

In general, the actuation mechanism of continuum robots [16, 17] can be divided into internal and external actuation mechanism [18]. Internal actuation mechanism refers to the robot actuator being located inside and as part of the ontology [19]. A typical example is pneumatic robots, where deformation is caused by the expansion of an internal elastic chamber [20]. External actuation mechanism refers to the use of external components to change the robot configuration, such as the magnetic continuum robot. Different actuation mechanisms would bring different characteristics to robots [21]. For instance, the magnetic robot is easy to miniaturize while the tendon-driven robot has a relatively large load capacity. In addition, even if the same design prototype is used, different degrees of manufacturing error will bring different model parameters to the robot. As time goes by, the wear effect will further damage the modeling accuracy of the robot.

Considering the nonlinear deformations caused by actuation, material elasticity, and sensitivity to contact with the environment, continuum robots face great challenges in precise analytical modeling. Although the kinematic modeling of traditional rigid linkage robots is completely defined by the size of linkage and joint coordinates, the almost unlimited freedom of continuum robots greatly increases the complexity of its modeling. One of the major challenges in modeling soft continuum robots is to simplify the models and compromise the relationship between computation complexity and model accuracy. At present, the main modeling methods of continuum robots include the continuum model, geometric model, and data-driven model [22]. In the continuum model, the continuum robot is represented by an infinite number of infinitesimal microsolids in continuous accumulation [23], with geometric accuracy. The geometric model assumes that the continuum robot deforms in a certain geometric form, and the piecewise constant curvature modeling method is the most commonly used assumption for the continuum robot at present [24]. Recently, data-driven modeling, in which the model of the system is derived by using datasets and learning processes without making such physical simplification assumptions, has also been widely studied [25–27].

The control problem for the continuum robot is to find the proper actuation value to reach the desired state to perform a given task. In the control field, most research works focus on the positioning control and force control of the end-effector, but the control of the whole body configuration is rarely studied [16, 28]. Due to the kinematic redundancy of the continuum robot, a hierarchical control strategy could be adopted to achieve the optimal control to perform a certain task and simultaneously achieve the optimal configuration of the body. Currently, the control methods could be mainly divided into model-based, model-free, and hybrid model control [29]. Among them, model-based control is highly dependent on the precise modeling of the continuum robot and the perception accuracy of the sensor, showing better performance in motion accuracy [30]. The model-free control method is a data-driven control method, which is based on the neural network to learn the model of the robot, so as to achieve efficient control [31]. The hybrid model control method is generally combined with the neural network and physical model, and the neural network model is used to compensate for nonlinear factors to achieve efficient control accuracy [32, 33]. In addition, remote operation is often used to control the continuum robot in the medical field [34, 35]. Through the user’s visual perception, with the help of intraoperative images, the robot’s state can be estimated, and the user can correct its movement accordingly.

This paper is aimed at summarizing and discussing the representative work in design, actuation, modeling, and control of continuum robots, as well as conducting a thorough and systematic analysis. This paper, in contrast to other review publications [21, 24, 36, 37], focuses more on major technical concerns rather than its application in a certain field. Thus, it could provide a relatively wide perspective on the development of continuum robots. Furthermore, this paper is expected to enlighten researchers to pay more attention to the immature field of continuum robots. This paper is organized as follows. In Chapter 2, this paper introduces the design and actuation method of the continuum robots in detail. In Chapter 3, this paper introduces the development of the continuum model, geometric model, and data-driven model in detail. In Chapter 4, several control methods of continuum robots, including model-based, model-free, and hybrid model control strategies, are introduced. In Chapter 5, some prospects and challenges are offered in order to motivate researchers to address some new issues. In Chapter 6, we conclude this paper and hope to give the researcher inspiration and a general understanding of continuum robots.

## 2. Design and Actuation Methods

### 2.1. Classification of the Design and Actuation Principle

Many alternative design and actuation approaches of continuum robots have been presented to fulfill the application in many sectors. Tendon-driven continuum robots are currently the most commonly used [38–40]. This kind of robots has a relatively rigid body [41], can easily obtain the analytical solution of the kinematics model, and is widely applied in multitask operation of various small surgical instruments [42]. Besides, it can usually achieve bending angles in excess of 100 degrees, achieving positioning accuracy of around 2.0 mm [43]. However, traditional tendon-driven continuum robots are constrained by traditional manufacturing processes and are difficult to be scaled down to smaller scales.

In order to reduce the stiffness of the continuum robot while maintaining a larger workspace, the fluid actuation mechanism [55–57] is proposed to replace the tendon actuation. Although its soft body gives the robot the advantages of safe contact and a large bending angle, it is difficult to achieve accurate positioning due to its highly nonlinear characteristics and the increasing complexity of the control system [58]. In addition, fluid-driven robots are larger than tendon-driven robots due to their limited actuation mode in shape.

In order to realize the miniaturization configuration of continuum robots, many cutting-edge materials have been developed and used to realize the actuation of robots, showing unique advantages [38, 59–61]. The soft continuum robots embedded with micromagnet or made of ferromagnetic composite material have accurate steering ability under an external controllable magnetic field; its bending angle could exceed 180 degrees and has high positioning accuracy up to 10 *μ*m [49]. Magnetically soft continuum robots, on the other hand, can achieve small diameters, up to the micron scale, which ensures their ability to conduct targeted therapy in bronchi or in cerebral vessels [62]. However, it is difficult for magnetically soft continuum robots to maintain stability under external forces, and the tiny rigid magnet tips risk falling off inside the body during operation. To achieve safer and more reliable control, shape memory materials are used to drive the continuum robot and for cardiovascular examination and nasopharyngeal administration [63]. The key advantage of this self-deforming material is that it provides extension, bending, and torsion for the main stem and can achieve overall actuation while maintaining a small scale, but its inherent hysteresis makes it difficult to achieve rapid response and precise positioning at the same time, and it has a low load capacity and quite complex pipeline wiring. Recently, combined with the ionic liquid conductors and tendon-driven method, a kind of continuum robot with proprioception has been studied. This robot show a promising prospect toward low-cost, scalable position feedback for small-scale continuum robots [64].

In order to achieve accurate position prediction and variable stiffness for different environments, researchers have developed a number of hybrid actuated continuum robots. By integrating the pneumatic and tendon actuation methods, the robot exhibited great characteristics and achieved bending angles greater than 90° [65, 66]. In order to achieve the interventional treatment of human stenosis, a continuum robot integrating magnetic- and tendon-driven methods is proposed [51]. The robot can achieve relatively large angle steering under tendon actuation and high-precision position control of 10 *μ*m under the external magnetic field. It takes the full advantage of both actuation methods and demonstrates excellent steering and accurate tracking capabilities.

Recently, some new design and actuation methods of continuum robots have been studied. A stretchable origami continuum robot [53] with omnidirectional bending and twisting has been developed. This kind of robot could achieve more sophisticated motions such as continuous stretching and contracting, reconfigurable bending, and multiaxis twisting with the foundation of the basic integrated motion. Besides, this robot has good scalability that could be assembled into a multisection continuum robot. A soft pneumatic robot [67, 68] has been studied that is able to navigate their environments not through locomotion but through growth. This motion is achieved by two principles: the increasing pressure of the thin-walled vessel allows rapid and substantial extension of the tip and asymmetric lengthening of the tip allows directional control. By validation, this kind of robot shows the abilities to navigate through constrained environments by exploiting passive deformations. The Table 1 summarizes some different design and actuation method of continuum robots.

**Table 1:** Summary of different design and actuation methods.

Literature	Design principle	DOF	Actuation	Diameter (mm)	Accuracy	Characteristics
[44]	Parallel	5	Rod driven	—	2.3%	Large workspace and high precision
[45]	Backbone	2	SMA driven	5	0.98 mm	Modular; teleoperation
[39]	Backbone	4	Tendon driven	38	<2 mm	Passive structural flexibility
[46]	Backbone	6	Fluid driven	30	—	Variable stiffness
[47]	Concentric tube	12	Motor driven	2.74; 1.92; 1.21	1%	Dual-arm
[48]	Backbone	4	Fluid driven	32	1.3%	Soft and has variable stiffness
[49]	Backbone	6	Magnetic driven	—	<7.86	Combination of soft and hard joints
[50]	Backbone	5	Magnetic driven	2.5	—	Variable stiffness
[51]	Backbone	5	Tendon and magnetic driven	3	10 *μ*m	Large workspace and high precision
[52]	Backbone	5	Fluid and tendon driven	25	—	High load capacity
[53]	Backbone	5	Magnetic driven	20	—	Omnidirectional bending and twisting
[54]	Concentric tube	4	Motor driven	4.36; 2.265	—	3D printed

### 2.2. Optimization Method of the Structure Parameters

For some specific occasions, the structure of continuum robots needs to be specially designed to meet the needs of use. Two important principles need to be considered: the size of the workspace and the stiffness. In general, the workspace and stiffness should be as large as possible, so that the robot could reach the desired position and apply the required operating force. The two properties mainly depend on the structural design and constituent material. In general, a small diameter and high elastic modulus would take a large range of workspace; however, small cross-sectional diameter would reduce the stiffness of the robots. Therefore, there is a basic compromise between the workspace and the stiffness of a continuum robot. The super-elastic NiTi skeleton has high stiffness in a small diameter (about 3 mm), which has been widely adopted as the backbone skeleton of robots [38, 69, 70]. Moreover, it has good biocompatibility and is widely used in the medical field.

In recent years, there have been some studies on the structure optimization of continuum robots [71, 72]. The optimization parameters include material properties, whole length, diameter, and curvature that affect robot capabilities and properties. It is significant to obtain optimal design parameters for concentric tube robot [73], considering surgical task requirements, anatomical constraints, and any other desired indicators. Heuristics algorithms have been proposed to solve the design optimization problem on workspace of the robots [74]. However, it has been proven that this method is actually suboptimal [75]. Recently, a systematic set of the evolutionary design algorithm by integrating a theoretical model and the genetic algorithm is proposed [76]. The experimental results validate that designed magnetic soft continuum robots have a counterintuitive nonuniform distribution of magnetic particles to achieve an unprecedented workspace. Besides, some researcher also appropriately integrates sampling-based motion planning in configuration space into stochastic optimization in design space to obtain the optimal continuum robot for medical application [77] and provide the asymptotic optimality.

## 3. Modeling Method

Many researchers have contributed to the development of mathematical modeling methods capable of describing the kinematics and dynamics of such robots with infinite degrees of freedom since the field’s inception. However, addressing the needs of robotics applications remains a challenge. The kinematics and dynamics modeling of the traditional rigid linkage robot can be defined by the size of the linkage and the coordinate of the joint. However, continuum robots have almost unlimited degrees of freedom, which greatly increases the complexity of modeling. In order to meet the standard of traditional rigid robot technology, the model of continuum robots should have low computational costs and sufficient accuracy. It should be able to clarify mathematical submodels, include them in a unified framework, and provide a systematic modeling process. At present, one of the main challenges of continuum robot modeling is to study and set up reasonable model assumptions and simplify modeling, which can accurately predict robot behavior while improving computational efficiency. The tradeoff between model complexity, computational cost, and accuracy is the main principle that must be considered when modeling continuum robots. This paper investigates the modeling techniques that have been proposed so far and introduces the development of the continuum model, geometric model, and proxy model, which are relatively representative in current academia.

### 3.1. Continuum Model

The continuum model is an infinite degree of freedom model in which a robot is represented by a continuous stack of infinite infinitesimal particles (Cosserat bar theory, see [78]). In the field of robotics, the Cosserat theory consisting of a finite number of solids projected on a continuous backbone has been applied to the dynamics of hyperredundant robots [79]. Recently, the Cosserat theory has been explicitly applied to soft robot motion and operation under static and dynamic conditions [79–81]. The Cosserat model treats the continuum robot as a deformable curve in which each particle is rigidly connected to a set of orthogonal vectors (controllers) to characterize its direction [82].

In Cosserat theory [79], the configuration of a microsolid with material abscissa $X\in \left[0,L\right]$ on the continuum robot with respect to the base frame of a continuum robot can be represented by position vector P and rotation matrix R. Therefore, the configuration space is defined as a curve $g\left(\cdot \right)\text{: }X\mapsto g\left(X\right)\in \text{SE}\left(3\right)$ and

(1)g=RP0T1.

The strain state of the soft arm is defined by the vector field along the curve $g\left(X\right)$ given as $X\mapsto \hat{\xi }\left(X\right)=g^{-1}\partial g/\partial X=g^{-1}g^{\prime }\in se\left(3\right)$ where the hat is the isomorphism between the twist vector representation and the matrix representation of the Lie algebra $se\left(3\right)$. It could be defined as

(2)ξ^=k~q0T0∈se3,ξ=kT,qTT∈R6,

where $q\left(X\right)$ represents the linear strains and $k\left(X\right)$ represents the angular strains.

The time derivation of the configuration curve $g\left(\cdot \right)$ is represented by the twisted vector field $X\mapsto \eta \left(X\right)\in R^{6}$ given by $\hat{\eta }\left(X\right)=g^{-1}\partial g/\partial t=g^{-1}\dot{g}$. This field can be detailed in terms of its components in the (micro)body frames as

(3)η^=w~v0T0∈se3,η=wT,vTT∈R6,

where $v\left(X\right)$ represents the linear velocity and $w\left(X\right)$ represents the angular velocity at a given time instant.

**Figure 1:**
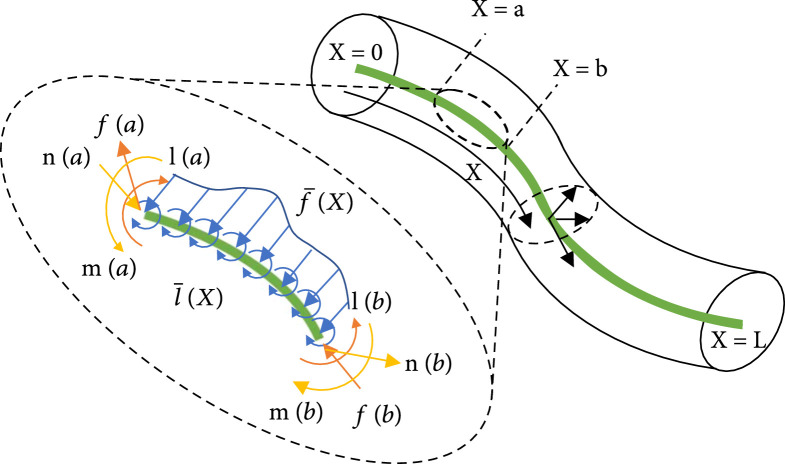
Diagram of force analysis of continuous media [83].

The time derivation of continuous media (see Figure 1) can be used to describe the dynamics that relates the time evolution of stress to the time evolution of strain. It could be obtained based on Newton’s law, Alembert’s law, and Hamilton’s law; a set of geometric boundary conditions; and the constitutive law as follows.

(4)Mη˙−adηTMη=Λ′−adξTΛ+F¯,Λ0=−F0,Λ1=F1,

where ad is the adjoint representation of the Lie algebra, M is the inertia tensor of the cross section, $\Lambda ={\left({\left(R^{T}m\right)}^{T},{\left(R^{T}n\right)}^{T}\right)}^{T},\bar{F}={\left({\left(R^{T}\bar{l}\right)}^{T},{\left(R^{T}\bar{f}\right)}^{T}\right)}^{T}$, and it simulates the stress field on the beam which is the double counterpart of the strain field.

In order to simplify the modeling complexity, a discrete Cosserat model based on piecewise-constant strain (PCS) hypothesis has been proposed in recent years [22]. Compared with finite element simulation and other methods, this model can express the motion characteristics of discrete beams by explicit integration. The strain assumptions of the Cosserat continuous model (including torsion, shear, curvature, and elongation) are considered. The discrete modeling framework has excellent modeling accuracy under different driving conditions and external load models and can represent the deformation under the interaction with the medium, which promotes the development of modeling theory in the field of continuum robots.

### 3.2. Geometric Model

Compared with the Cosserat model, the geometric model does not consider the material properties of the robot and assumes that the deformation of the robot is a specific geometric shape. Therefore, the configuration of the continuum robot can be represented by a curve and a vector defining the direction of the robot tip. By far, the most widely used geometric model in the soft robot field adopts the piecewise constant curvature assumption [24]. The soft robot is expressed as a finite set of arc and described by circle parameters (radius of curvature, arc angle, and bending plane). By this method, the dimension of the state vector of continuum robot is simplified, and it is widely used in all kinds of real-time control algorithms and other occasions requiring high-speed computation [84–86].

**Figure 2:**
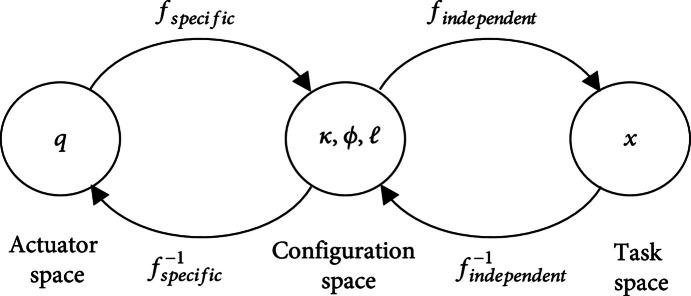
Mapping relationship of geometric model.

As shown in Figure 2, the kinematics of the robot is decomposed into two mappings: One mapping is $f_{\text{specific}}$ from the joint or actuator space q to a configuration space parameter $\left(\kappa ,\varphi ,l\right)$ describing a constant curvature section; it is robotic specific because each actuator in a unique continuum robot would affect circle parameters in different ways. The other mapping $f_{\text{independent}}$ is from the configuration space to the task space; it is robot independent and used to represent the characteristics of piecewise constant curvature deformation.

**Figure 3:**
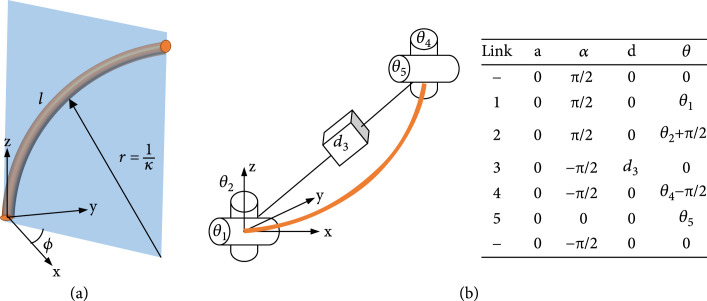
Parameter description of the configuration space continuum robot. (a) Arc parameter description of one section of continuum robots. (b) Denavit-Hartenberg (D-H) parameter description of one section of continuum robots.

As is shown in Figure 3(a), the continuum robot can be described by a spatial curve, which describes the position and direction of any point on the body. Kinematic models of robots can be derived in a variety of ways, including Denavit-Hartenberg (D-H) parameters [87], Frenet-Serret (F-S) framework [88], and exponential coordinates [89]. At present, the kinematic characterization method using the DH parameter and exponential coordinate is widely used. In the section, we mainly introduce the two-representation method.

As shown in Figure 3(b), the continuum robot can be represented as the relative rotation and translation motion between multiple rigid links, and the corresponding DH parameters are given. According to the assumption of constant curvature deformation, the transformation matrix $T_{c}$ between the tip coordinate system and the base coordinate system can be represented as

(5)Tc=cos2φcosκs−1+1sinφcosφcosκs−1cosφsinκscosφ1−cosκsκsinφcosφcosκs−1cos2φ1−cosκs+cosκssinφsinκssinφ1−cosκsκ−cosφsinκs−sinφsinκscosκssinκsκ0001.

Similar results can be obtained using exponential coordinates based on the Lie group theory [90, 91]. The homogeneous transformation of the constant curvature curve can be decomposed into rotation transformation φ and in-plane transformation ℓ, and we can write the twist vector associated with each transformation using the notations and conventions outlined by Murray [92] as follows.

(6)xrot=vrotwrot=0 0 0 0 0 1T,xinp=vinpwinp=0010κ0T.

It can be written as follows.

(7)x^rot=w^rotvrot00=0−100100000000000,x^inp=w^inpvinp00=00κ00000−κ0010000T.

Using the exponential product formula, the transformation matrix T could be solved by the following equation.

(8)T=ex∧rotφex∧inpℓ.

Similarly, if a set of twist vector is denoted by

(9)x=001−κsinφκcosφ0T.

Its exponential coordinate has the same form with (5).

### 3.3. Data-Driven Model

Because of the significant nonlinearities in the motion process of continuum robots, successful modeling of robots necessitates a significant amount of work and experience in continuum mechanics. To tackle these challenges, data-driven techniques, in which systems are modeled by learning from vast volumes of data (inputs and outputs) from external sources, have gotten a lot of attention recently [93–96]. At present, the neural network (NN) is the most commonly used approximate mapping regression model. By weighting the hidden layer, information always flows from the input to the output [97]. Furthermore, various regression approaches have shown to be effective in the field of continuum robotics, and the representative method can be locally weighted projection regression (LWPR) and (local) Gaussian process regression (GPR) [98]. One of the main benefits of these solutions is that they do not require physical models, but they rely on large amounts of representative data, which is sometimes difficult to collect. It is worth noting that for both data-driven and model-based approaches, each has a unique strength and is preferred over the other in certain scenarios. Due to the possibility of hybrid approaches, combining both of them should also be explored in the future. Neural network models, on the other hand, are often pretrained and difficult to adapt to dynamic contexts. As a result, online learning is likely to become a new research topic.

## 4. Control Method

Robot control is the study of how to determine the right amount of drive to achieve the required state to perform a given task. The state of the continuum robot includes the position and orientation of the end-effector, the configuration of the robot, the stiffness of the robot, and its related motion performance. Current control methods of continuum robots mainly focus on achieving accurate position and posture control of end-effector and impedance control of the end-effector [16, 28, 99–101], which are mainly solved at the level of actuation and design optimization. There are few studies on control methods of overall configuration and distributed stiffness. On the other hand, the continuum robot has a redundant degree of freedoms, and the optimal control method can be used to achieve the optimal configuration of its configuration while performing the required tasks [102, 103].

Currently, control methods for the continuum robots can be divided into model-free, model-based, and hybrid model controls [29]. The accuracy of the robot model is critical for model-based control approaches. In order to obtain satisfactory control effects, complex models are usually derived to explain the numerous physical phenomena experienced by the robot. It is challenging to implement high-performance control algorithms in real time when using sophisticated nonlinear models, such as the variable curvature model. Control accuracy can be improved by using electromagnetic sensors [104, 105] or analytical calculation [106], visual feedback [107, 108], and other feedback techniques. The measured data is used to compensate the model error in the control process and achieve higher control accuracy. A typical closed-loop control strategy for a magnetic continuum robot is proposed by Campisano et al. based on the real-time Cosserat rod theory [109]. It utilized the actuation feedback to compensate for nonlinearities that can result in a kinematic model error. Pose feedback is utilized to maintain accurate path following. Experimental results demonstrate that the closed-loop control scheme has a significant performance.

Several studies have shown that using a closed-loop control strategy with feedback information can significantly increase control accuracy, stability, and robustness in low-frequency environments [110–113]. However, for extremely miniaturized continuous robotics, feedback control poses significant technical problems in sensor integration. It incorporates compatibility with anatomy, preoperative imaging technologies, and surgical equipment, especially in medical applications [105, 114]. Contact constraints in the environment also bring uncertainty to the control of the continuum robots [26, 37]. In order to overcome the inaccuracy of the model, some advanced control methods, such as adaptive control, have emerged in the field of continuous robots. The earliest application of this method was to compensate the kinematic inaccuracy of concentric robots [115] and to realize the automatic adjustment of model parameters by using model parameter estimation, so as to achieve high-performance control [116].

**Figure 4:**
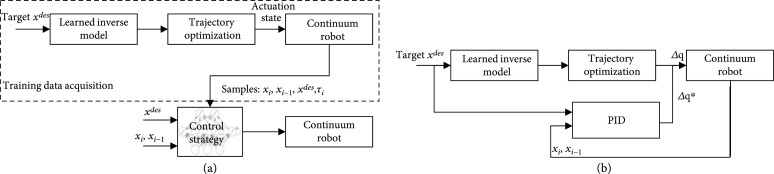
Several control strategies for continuum robots. (a) Schematic diagram of learning-based control strategy. (b) Schematic diagram of hybrid control strategy.

Due to the high complexity and high computation time of continuum robot kinematics, the model-free method (see Figure 4(a)) is used as an alternative method in recent studies. Direct strategy learning for robot control is an effective method, which is suitable for situations where dynamic modeling is difficult or the environment is unstructured. This method can be applied to high-dimensional systems and has a higher speed than traditional model-based controllers [93, 120, 121]. Strategy learning can be divided into model-free reinforcement learning and model-based reinforcement learning; we focus on model-based reinforcement learning because it can generate more effective strategy learning samples. In [122], the model-based strategy search uses a learning control algorithm called probabilistic reasoning, which takes into account the model uncertainty of the learned dynamic model (provided by a nonparametric Gaussian process) in long-term planning. Recently, there has been a strong interest in using traditional trajectory optimization methods to generate samples for strategy learning [123]. In addition, it can also combine the function approximation ability of the neural network to learn and represent these strategies [124, 125]. The most advanced methods of using this variant of the idea involve the use of local models of learning [126], composite multistep controllers [127], and deep representations of control strategies [128]. A model-based policy learning algorithm is proposed for closed-loop predictive control of a continuum robot. The closed loop control is obtained by trajectory optimization and supervised learning strategy, which shows good performance on control accuracy.

However, high computational time, low compatible rate of change in environment/interference, and complexity of learning methods (due to the nonlinearity and redundancy of continuum robots) seriously hinder the use of model-free methods in complex scenarios. The adaptability of different continuum robot structures presents additional challenges to the learning approach due to the particularity of each structure and inconsistencies in actuation and model descriptions. Therefore, a hybrid model control method (see Figure 4(b)) is proposed, which takes into account both model control reliability and data-driven robustness. One representative work is the hybrid adaptive control framework proposed by Wang et al. [116]; it combined offline trained robot inverse kinematics with neural network and online adaptive PID controller to compensate the positioning error caused by external disturbance. The experiment results validated that the proposed hybrid adaptive control framework has great performance to compensate for uncertain factors such as friction, driving tendon relaxation and external load during robot movement. Another representative work is the control method based on the Koopman operator theory [119, 129], which can not only avoid the physical simplification hypothesis but also produce a clear control-oriented model. This method uses the linear structure of the Koopman operator to construct a linear model of a nonlinear controlled dynamical system from input-output data and uses an established linear control method [130, 131] to control it. Koopman modeling and control methods are well suited to soft robots because they pose less physical threat to themselves or their surroundings, which allows the model to safely collect input-output data under a variety of operating conditions and do so in an automated manner. In addition, because the Koopman program is entirely data-driven, it inherently captures input-output behavior, avoiding the ambiguity involved in selecting discrete sets of states for structures with infinite degrees of freedom [119]. The Table 2 summarizes some different control methods for continuum robots.

**Table 2:** Summary of different control methods.

Literature	Design principle	Actuation	Model based or free	Control method	Accuracy
[116]	Backbone	Tendon driven	Model based	Hybrid control based on the adaptive PID and NN model	<2.14 mm
[26]	Backbone	Tendon driven	Model free	Optimal control considering contacts	—
[110]	Backbone	Tendon driven	Model based	Closed control based on the mechanics model	<1.07 mm
[111]	Backbone	Magnetic driven	Model based	Closed control based on the mechanics model	0.42 mm
[117]	Backbone	Tendon driven	Model based	Open control based on the FEM model	<2.14 mm
[112]	Backbone	Fluid driven	Model based	Closed control based on the inverse kinematics	12.3 mm
[113]	Concentric tube	Motor driven	Model based	Closed control based on the Cosserat rod model	<1.67 mm
[118]	Backbone	Magnetic driven	Model based	Closed control based on the inverse kinematics	<5.41 mm
[93]	Backbone	Tendon driven	Model based	Closed control based on the reinforcement learning	<0.029 mm
[119]	Backbone	Fluid driven	Model based	Closed control based on the Koopman operator theory	<0.43 mm
[120]	Backbone	Fluid driven	Model based	Closed control based on the online learning kinematic	16.8 pixels

## 5. Prospects and Challenges

In this paper, we have reviewed the state of the art of continuum robots, focusing particularly on the design, actuation, modeling, and control. The design and actuation of continuum robots is evolved from single actuation to hybrid actuation combining the advantages of different actuation methods. On the other hand, some new type of continuum robot including soft pneumatic growing robot or origami-inspired continuum robot shows excellent abilities in motion flexibility or expansibility. On the field of modeling, the most widely used method includes Cosserat model and geometric model. The former is geometric exact but has low computation efficiency. The latter simplifies the modeling complexity and has very high computation efficiency but has lower accuracy if there is large deformation. However, considering the requirements of many applications, the geometric model is still popular. Recently, the data-driven method has been studied and applied into the modeling of continuum robots due to its ability of nonlinear fitting and generalization. However, the generalization ability of dynamic scenarios is still a problem worth studying. On the field of control, the closed control strategy with feedback information shows great improvement on the control accuracy, stability, and robustness under low frequency environment; however, it depends mostly on the sensor accuracy. Recently, the model-free control method which is used as an alternative method has been studied. Direct strategy learning for robot control is an effective method and shows good control accuracy and robustness. However, these methods have low adaptability to the change in environment/interference. The hybrid model control method attracts the attention of researchers. The Koopman algorithm uses the linear structure of the Koopman operator to construct a linear model from input-output data to describe the dynamics of a complex system. Thus, many linear model controllers could be applied to the motion control of some complex continuum robot.

However, there are some serious significant challenges for the development of continuum robots, and there is no good solution so far.

(1) The first is the miniaturization of continuum robots. At present, only magnetic-driven, optical-driven, or thermal-driven methods are most likely to achieve the miniaturization of continuum robots, but they often have highly nonlinear complex mechanical models, which makes it difficult to achieve robust control
(2) The second is to enhance continuum robot perception. Ideally, the optimal awareness should be able to reconstruct the configurations of the robot with infinite degrees of freedom; however, only expensive FBG optical fibers can currently realize a high degree of freedom reconstruction. In the future, it is expected to combine with optical, mechanical, and electrical technologies to develop sensors that are cheaper and more suitable for continuum robots
(3) The third is to develop the physics simulation engine of the continuum robots. The traditional rigid robot has a number of relatively mature simulation frameworks; however, they are not suitable to the continuum robots with infinite degrees of freedom. Nowadays, the validation of control algorithms must depend on the real robot platform which increases the costs. It is urgent for academia and industry to develop a real-time physics simulation engine for soft continuum robots

## 6. Conclusion

In summary, the development of continuum robots is rapid in recent years, and there have been many representative achievements on the design, actuation, modeling, and control of continuum robots. These great improvements promote the application of continuum robots in many fields such as surgical robot, nursing robot, continuum-limbed vehicles, ship-to-ship refueling, and exploration of extraterrestrial surfaces. It is expected that the continuum robot would play a more and more important role in social production and human life.
